# CASMI: And the Winner is ..

**DOI:** 10.3390/metabo3020412

**Published:** 2013-05-24

**Authors:** Emma L. Schymanski, Steffen Neumann

**Affiliations:** 1 Eawag: Swiss Federal Institute of Aquatic Science and Technology, Überlandstrasse 133, CH-8600 Dübendorf, Switzerland; 2 IPB: Leibniz Institute of Plant Biochemistry, Department of Stress and Developmental Biology, Weinberg 3, DE-06120 Halle (Saale), Germany

**Keywords:** mass spectrometry, metabolite identification, small molecule identification, contest, metabolomics, non-target identification, unknown identification

## Abstract

The *C*ritical *A*ssessment of *S*mall *M*olecule *I*dentification (CASMI) Contest was founded in 2012 to provide scientists with a common open dataset to evaluate their identification methods. In this review, we summarize the submissions, evaluate procedures and discuss the results. We received five submissions (three external, two internal) for LC–MS Category 1 (best molecular formula) and six submissions (three external, three internal) for LC–MS Category 2 (best molecular structure). No external submissions were received for the GC–MS Categories 3 and 4. The team of Dunn *et al.* from Birmingham had the most answers in the 1*^st^* place for Category 1, while Category 2 was won by H. Oberacher. Despite the low number of participants, the external and internal submissions cover a broad range of identification strategies, including expert knowledge, database searching, automated methods and structure generation. The results of Category 1 show that complementing automated strategies with (manual) expert knowledge was the most successful approach, while no automated method could compete with the power of spectral searching for Category 2—if the challenge was present in a spectral library. Every participant topped at least one challenge, showing that different approaches are still necessary for interpretation diversity.

## 1. Introduction

Mass spectrometry has become one of the most important analytical techniques in both the environmental sciences and metabolomics. The potential of untargeted approaches is increasing rapidly with the recent advances in high accuracy mass spectrometry and the result is an explosion in the number of options available to process data and identify compounds, but few systematic comparisons of these different approaches exist. With the sheer number of options available, it is impossible to evaluate all programs oneself.

With CASMI, the *C*ritical *A*ssessment of *S*mall *M*olecule *I*dentification, we initiated an open contest to let the experts showcase their own programs and strategies, so that users can compare the results and choose the strategies that apply to them best. Since the experts and programmers generally know their own settings best but have access to different data, CASMI addresses this by providing one common set of data. CASMI was inspired by CASP, the *C*ritical *A*ssessment of (protein) *S*tructure *P*rediction contest series initiated in 1994 [1,2].

We set up a website [3] to publish spectral information for a set of known compounds that were unknown to the participants, along with some background information to help with the identification, where available. We then called on the mass spectrometry community to propose identities for the unknowns. We introduced four categories, two for liquid chromatography coupled with high accuracy (tandem) mass spectrometry (LC–HRMS/MS) and two for unit resolution gas chromatography–mass spectrometry (GC–MS) data. There were 14 challenges for Categories 1 and 2 (best molecular formula and best structural formula, respectively, for LC–HRMS/MS) and 16 challenges for Categories 3 and 4 (best molecular formula and structural formula, respectively, for GC–MS). The challenges and categories are discussed in detail in the “CASMI: Challenges and Solutions” article within this special issue [4], which also includes annotated spectra of the LC–HRMS/MS challenges. A summary table including the 14 LC–HRMS/MS challenges is given in Table A1.

### 1.1. Background of the Inaugural CASMI

The idea to found CASMI came up in early 2012, along with the opportunity to guest edit a special issue of *Metabolites*. Although this was a somewhat “backwards” start (with the proceedings in place before the competition even existed), the rest fell into place quite quickly. The organisation team consisted of one representative from bioinformatics/metabolomics (S. Neumann) and one from environmental chemistry (E. Schymanski), with the aim of bringing both (and additional) communities together to improve the exchange and learn from each other’s methods. An advisory board was also formed, consisting of four members: V. Likic (founding Editor-in-Chief of *Metabolites*), S. D. Richardson (US EPA), S. Perez Solsona (CSIC, Spain) and L. Sumner (Noble Foundation, US). Participants were recruited via email, social media, public announcements at several meetings (including SETAC, ASMS, IMSC and in workshops; see [5] for more details) and finally in a Spotlight article in MetaboNews [6]. Mailing lists were available to participants to sign up for announcements and discussion.

The key dates for CASMI were:

20/05/2012: Public release of www.casmi-contest.org
20/07/2012: Public release of challenge data
31/01/2013: Deadline for submissions (extended to 05/02/2013)
06/02/2013: Public release of solutions
22/02/2013: Public release of automatic evaluation (07/03/2013 for resubmissions)

In this review we first outline the contest rules and evaluation measures, then describe the participants, their methods and submissions. We then discuss the results by challenge and conclude with some perspectives for future CASMIs. Details on the challenges and solutions are given in a separate paper [4]; a table containing the LC–HRMS/MS challenges is given in Appendix A.

## 2. Methods: Evaluation and Ranking of Participants

It was obvious to us already while establishing the rules for CASMI 2012 that it would not be possible to establish only one, simple evaluation measure to compare the performance of the participants. There was already sufficient variety in the scoring systems of our own methods to get a feel for the flexibility that would be needed, and we had to be prepared for many cases that we could not anticipate in advance. In this section we describe the measures we used and their advantages and disadvantages, using data from the CASMI contest. Although we only needed to use the absolute rank of the correct solution to declare the winners in the end (see below), we still present all evaluation methods in this review.

### 2.1. Absolute Ranking

An *absolute ranking* is the simplest measure of evaluating entries, and this is what we used to declare the winners of the CASMI contest in the end. In real identification efforts, one needs to know how many “incorrect” solutions are placed higher than the correct solution. Although at the first glance an absolute rank appears simple, the devil is in the details, e.g., if several candidates have an identical score. A “best case” absolute ranking will look better, but is overly positive and ignores the fact that several candidates with equal scores existed. The “worst case” rank is rather pessimistic, but represents the situation more realistically as all candidates with equal scores will need to be considered in identification efforts. Although compromise values such as the average of the two could be calculated, these have little meaning in real life and were not considered further. As a result, we used worst case rank in our evaluation, as follows:

*Rank_WorstCase_* = *BC* + *EC*

where BC and EC stand for the number of candidates with *B*etter and *E*qual scores, respectively. If the score of the correct candidate is unique, then *EC* = 1.

The absolute rank was calculated by sorting the submissions by score and then searching for the position of the correct answer, as well as the number of candidates with equal score. The correct molecular formula for Categories 1 and 3 was identified using a straightforward string comparison. To avoid problems with different notation systems, all molecular formulas were first normalised using the R package Rdisop [7,8].

The comparison of structural candidates for Categories 2 and 4 was more complicated. We accepted two different structure representations, the standard InChI [9,10] or the SMILES code [11]. A simple string comparison of SMILES could easily miss the correct solution, because many valid SMILES are possible for the same molecule. Thus, the structure representations in the submissions were converted to the InChI Key during the evaluation using OpenBabel version 2.3.0 [12]. The first 14 letters (the first block) of the InChI Key describe the molecular connectivity or skeleton. The second block contains 8 letters describing the stereochemistry, tautomerisation and isotopes. Two additional letters provide information about the version of InChI (conversion) used and the last letter indicates the charge state. Although designed to be nearly unique, identical InChI Keys are possible for two totally different InChI strings, but this is very rare [13]. As mass spectrometry cannot generally determine stereochemistry (there are exceptions but this was beyond the scope of the current CASMI), we considered a structural candidate to be correct for CASMI if the first block of the InChI Key was identical to that of the correct solution. Where candidates with different stereochemistry were present, we took the match with the highest score to determine the rank.

The winner(s) of a single challenge were those participants who achieved the best absolute rank. The overall winner of a category was then the participant who achieved the most wins, based on their original submissions.

The disadvantage of absolute ranking is that it does not take the number of candidates into account. If two participants both have the correct solution at rank 50 and one candidate list contains 100 candidates, while the other contains 1000, the latter is certainly the more selective of the two, although the absolute result is the same. The selectivity can be assessed using relative ranking.

### 2.2. Relative Ranking

The *relative ranking position* (*RRP*) is a measure of the position of the correct candidate relative to all the other candidates and is shown in the equation below. As higher scores are inherently considered “better” than low scores, we used the *RRP* defined in, e.g., [14], such that *RRP* = 1 is good and *RRP* = 0 is not. For each submission, the total number of candidates (*TC*), the number of candidates with a better score than the correct structure (*BC*) as well as the candidates with an equal (*EC*) and worse score (*WC*) were used to calculate the RRP as follows:

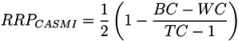

This *RRP* is only defined where *TC* ≥ 2 and also cannot be calculated for cases where the correct solution is absent. If the solution was present and all candidates have the same score, then *RRP* = 0.5. The relative ranking is a compact way to represent how well the candidate selection (scoring) performs for large and variable result sets, demonstrated with a few examples from CASMI 2012 here. Details on the participants are given below in Section 3.

− For Challenge 1, Category 2, Shen *et al.* have *TC* = 6, *BC* = 4, *WC* = 1 and a *RRP* of 0.20; while Ruttkies *et al.* have *TC* = 1423, *BC* = 21, *EC* = 24, *WC* = 1378 and a much higher *RRP* = 0.98, although the absolute rank of 45 is worse than Shen *et al.*’s 5;
− For Challenge 1, Category 2, Oberacher and Dunn *et al.* have *TC* = 1 and the *RRP* is undefined (both were correct), while Gerlich *et al.* have *TC* = 1356, *BC* = 0, *WC* = 1355, with *RRP* = 1.00; here Oberacher, Dunn *et al.* and Gerlich *et al.* share the honours, which is not visible from the RRP.
− For Challenge 12, Category 2, all participants missed the correct answer; the *RRP* is undefined.

While the *RRP* has obvious strengths, namely putting the absolute rank into perspective where hundreds or even thousands of candidates need to be considered, one potential problem is that a participant could “cheat” by not pruning irrelevant candidates from the end of the list, thus inflating the *RRP*. In other words, the *RRP* can also put participants with only a few high quality candidates in disadvantage. This can in turn be addressed by weighting this relative ranking with the candidate scores.

### 2.3. Normalised Scores and Weighted RRP

Most identification methods use some form of scoring to rank the candidates. In order to compare the different scoring schemes of the participants, we normalised all scores 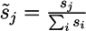 in a submission such that ∑*_i_s̃_i_* = 1 and calculated a *weighted RRP* using the score *s̃_j_* of the correct solution to obtain the sum of all better-scoring candidates 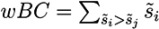 and equivalently 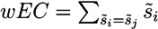 to give:

*wRRP_CASMI_* = 1 − *wBC* − *wEC*

The following examples from CASMI submissions help to interpret these values:

− For Challenge 10, Category 2, Ruttkies *et al.*, Gerlich *et al.* and Meringer *et al.* had the correct solution with absolute ranks 302, 307 and 63, respectively. The *wRRPs* of 0.01, 0.35 and 0.49 show that the scoring used by the latter two were more useful than the first in selecting the correct candidate, however given normalised scores (*s̃*) of ≤ 0.01 for all three, few users would have considered these candidates;
− For Challenge 2, Category 2, Shen *et al.* and Gerlich *et al.* have *wRRPs* of 1.00 and 0.91 respectively.
− For Challenge 13, Category 1, Dührkop *et al.*, Neumann *et al.* and Meringer *et al.* all have rank = 1, *RRP* = 1.00 and *wRRP* = 1.00, with *TC* = 20, 141 and 10, respectively. The normalised scores of 1.00, 1.00 and 0.14, respectively, indicate that the scoring system of Dührkop *et al.* and Neumann *et al.* weight the top candidate heavily—which is advantageouswhen the top candidate is correct, but can offer a false sense of security when interpreting the results.

The normalisation thus gives good results for those with few candidates and a very wide range in scores if the correct solution is in the top ranks, while those who have many candidates whose scores differ relatively little suffer from low normalised scores.

### 2.4. Similarity Between Submissions and the Correct Solution (Category 2)

The remaining question in the evaluation was assessing the chemical similarity between the candidates submitted by participants and the correct solution. This allows us to also assess how close (or misleading) the better candidates were, and how close the contestants were who missed the correct solution. Participants reliant on database entries are unable to identify the correct molecule if it is not in any spectral library or compound database, but they could still get very close. There is also a difference between a contestant who, e.g., obtained the wrong formula and thus also completely incorrect structural candidates and a contestant who reported the wrong positional isomer of the correct compound.

In the case of chemical structures, it is possible to calculate the similarity between any candidate structure and the correct solution. A common approach is to generate the “fingerprints” of two different molecules and compare these to come up with a similarity measure. We used the extended binary (1024 bitset) fingerprint calculation from the Chemistry Development Kit (CDK) to determine the fingerprint bitsets for candidates [15,16]. The Tanimoto similarity (*TS*) was then used to compare the bitsets [17,18]. As only the bits of value 1 are considered relevant in this comparison, we can define A and B as the number of bits equal to one in each bitset and C as the number of common bits equal to one in both bitsets. The *TS* is then:

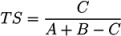

with a value between 0 and 1. The following simple example shows the CDK fingerprint bitsets for ethanol and ethane, respectively, and the resulting Tanimoto similarity.

[301 638 742 743 930]; [638 743]; *TS* = 0.4

A similarity measure of 1 corresponds to compounds with the same fingerprint, typically identical or very similar structures. Alternative fingerprints and distance functions are available; our choice was directed mainly by accessibility in the evaluation framework we used (see Section 2.5).

**Figure 1:**
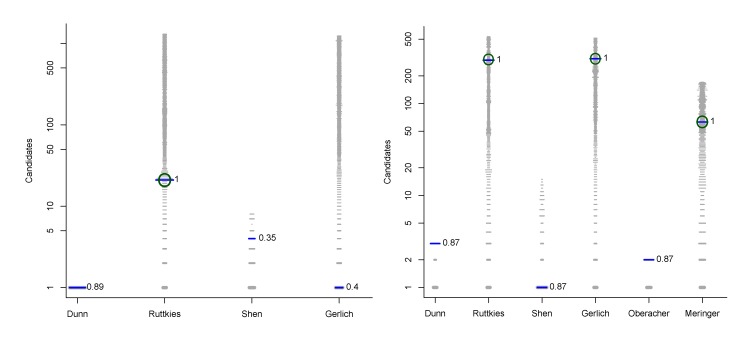
Similarity calculations for Category 2 entries for Challenges 3 (left) and 10 (right). Each candidate is represented by a grey dash, scaled by the similarity between candidate and the solution. The green circle indicates the correct answer if present; the blue dash indicatesthe most similar compound, with the *TS* adjacent. *y*-axis: number of candidates (log scale); *x*-axis: participant.

We used the similarities to compile plots of all (Category 2) entries from each contestant, with a dash for each candidate, where the length of the dash corresponds to the similarity with the correct solution. By marking the most similar molecule and the correct answer (where present), it is thus possible to assess quickly where the correct answer lay within the list of candidates (sorted by score) and also, for those who missed the correct answer, which was the most similar entry. The resulting similarity calculations thus allow us to assess which contestant was the “closest” if all contestants missed. We demonstrate this here using real examples from the participants; details about the participants are below in Section 3. Example plots for Challenges 3 and 10 are shown in Figure 1.

**Figure 2:**
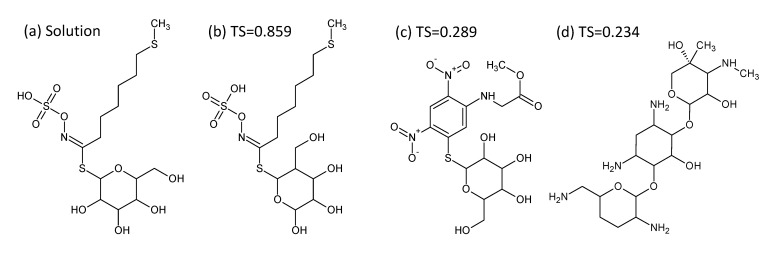
Most similar entries for Challenge 3. (**a**) The correct answer from Ruttkies *et al.*; (**b**) most similar entry from Dunn *et al.*; (**c**) most similar entry from Gerlich *et al.*; (**d**) most similar entry from Shen *et al.*; *TS* = Tanimoto similarity.

− In Challenge 3, Category 2, the entry from Ruttkies *et al.* contained the correct compound, while the other three contestants in this category missed it. The correct answer and TS with the most similar entries from the other participants are shown in Figure 2. This figure and the similarity scores show that Dunn *et al.* were the closest of the three contestants that missed.
− In Challenge 10, Category 2, Dunn *et al.*, Shen *et al.* and Oberacher all missed the correct solution, 1-aminoanthraquinone. However, all entries contained the positional isomer 2-aminoanthraquinone, in third, first and second place (by score), respectively. The Tanimoto similarity between the two positional isomers is 0.842.

This similarity measure provides important information in the absence of the correct solution and allows a quick assessment of whether the submission contained very similar structures or a very mixed set of structures. If the submission contains only very similar structures, it may be possible to deduce the compound class or a *maximum common substructure* of the unknown. This would equate to a “Level 3” identification (putatively characterised compound class) in the proposed minimum reporting standards in metabolomics [19]. This idea has also been applied recently in various ways, including “prioritising” candidates for MetFrag [20] and even defining substructures for structure generation [21]. If the candidates include many diverse structures, this task is more difficult and not even a compound class can be proposed.

### 2.5. Evaluation Framework

All evaluations and the corresponding graphics were performed with a set of scripts based on the statistics framework R [22], using extension packages including rCDK [23] and Rdisop [7]. The summary tables were created using xtable and reshape2, which allowed us to publish the results of the automatic evaluation rapidly. The evaluation script is available from the CASMI web site [24].

## 3. Results by Participant

In this article, we will refer to the participants by their surnames rather than their methods, as this enables us to associate a consistent, short description with each submission (as not all methods have a short, descriptive name). In this section we describe the methods briefly; the participants have described their methods more extensively in the individual papers submitted as part of this special issue [25,26,27,28,29,30].

### 3.1. Participation Rate

The individual results for each category, challenge and participant are available on the CASMI website [31]. Three external participants took part in Category 1 (Dunn *et al.* [25], Shen *et al.* [26] and Dührkop *et al.* [27]). As we had processed the challenges with our own methods to test the evaluation, we also submitted these results as two “internal participants” (Neumann *et al.* [29] and Meringer *et al.* [30]).

Three external participants entered Category 2, Dunn *et al.* [25], Shen *et al.* [26] and Oberacher [28]. As for Category 1, we submitted entries using our own methods as three internal participants, Ruttkies *et al.* [29], Gerlich *et al.* [29] and Meringer *et al.* [30]. No external participants submitted results for Categories 3 and 4 and the internal submission is presented on the CASMI website for completeness. Although this is not discussed in this article, some discussions can be found in [30].

The lack of participants can be narrowed down to two main reasons. The first was that our assumption that all methods could read an open data format (such as plain text peak lists) was incorrect and some systems only read native formats—which cost us at least one participant. It is imperative for the benefit of researchers, scientists and users alike that all systems can deal with at least one open format, not only to ensure transparency in the methods, but also to allow users to mix and match methods. The time requirements for both the submissions and the (optional) contribution to the special issue also discouraged potential participants. However, the participants we had covered a wide range of methods and provided plenty of interesting results, as discussed below.

The first CASP contest in 1994 had 33 challenges, 35 participants and 100 submissions in total. We had fewer external participants (4 in total) but received a total of 30 and 39 official submissions for the 14 LC–HRMS/MS challenges in Categories 1 and 2, respectively, which approaches the submission number for the inaugural CASP. Counting our internal submissions, we topped the number of CASP submissions easily, with 130 submissions in total for the LC–HRMS/MS challenges. Thus, we are optimistic that CASMI will grow into an established initiative like CASP.

### 3.2. Summary Results by Participant

**Figure B1:**
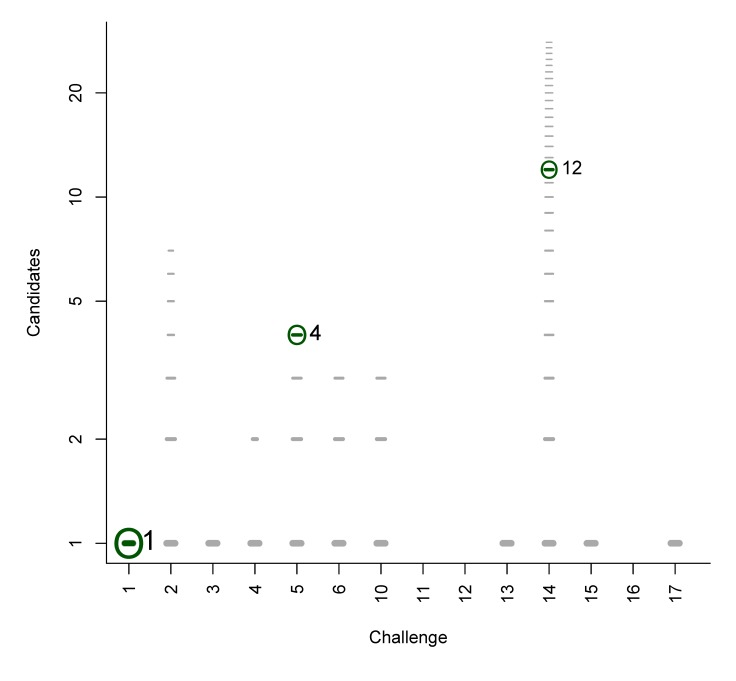
Category 2 entries of Dunn *et al.*: Grey dashes represent candidates, scaled by the normalised score. Green circle and dash represent the correct entry (when present), with the rank written adjacent.

**Figure B2:**
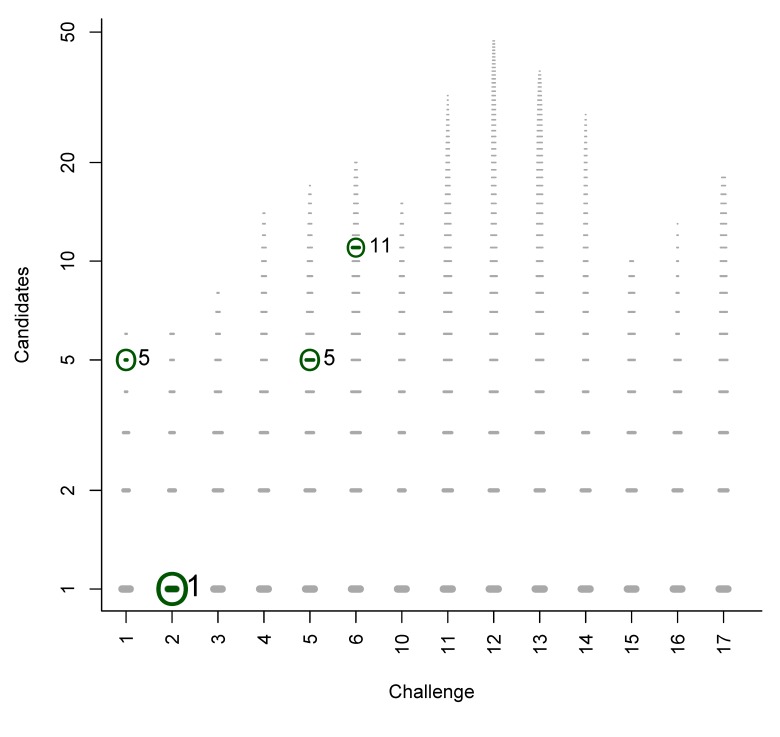
Category 2 entries of Shen *et al.*: Grey dashes represent candidates, scaled by the normalised score. Green circle and dash represent the correct entry (when present), with the rank written adjacent.

**Figure B3:**
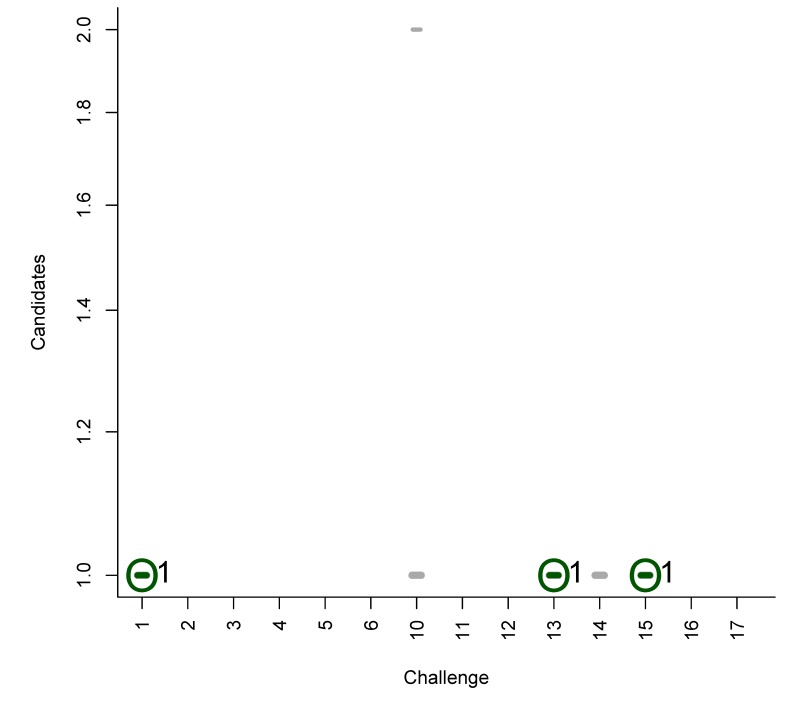
Category 2 entries of Oberacher *et al.*: Grey dashes represent candidates, scaled by the normalised score. Green circle and dash represent the correct entry (when present), with the rank written adjacent.

**Figure B4:**
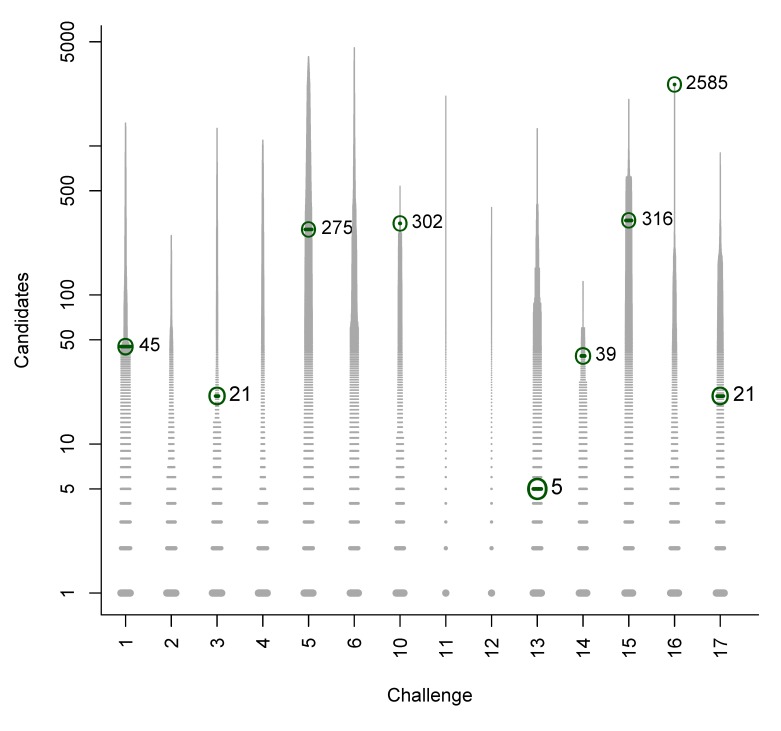
Category 2 entries of Ruttkies *et al.*: Grey dashes represent candidates, scaled by the normalised score. Green circle and dash represent the correct entry (when present), with the rank written adjacent.

**Figure B5:**
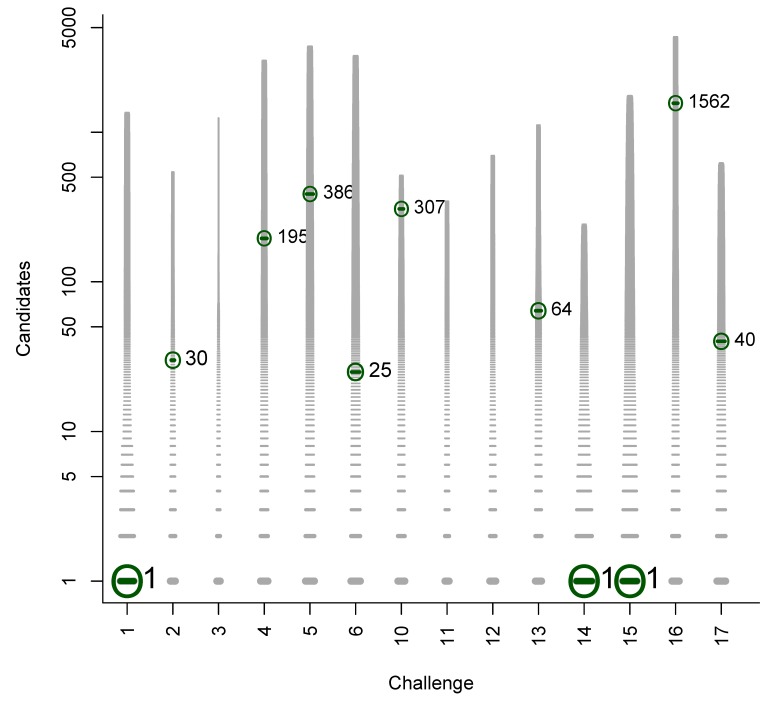
Category 2 entries of Gerlich *et al.*: Grey dashes represent candidates, scaled by the normalised score. Green circle and dash represent the correct entry (when present), with the rank written adjacent.

**Figure B6:**
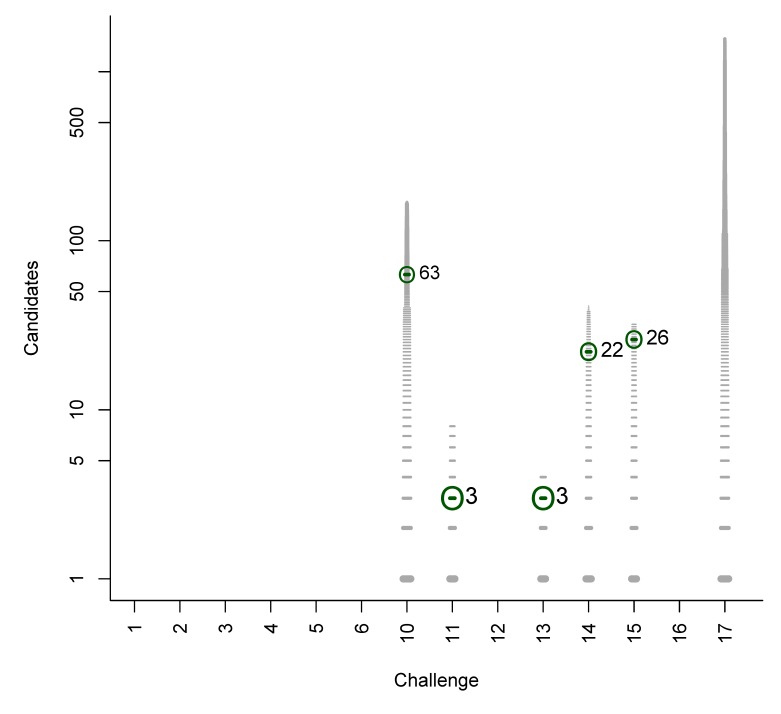
Category 2 entries of Meringer *et al.*: Grey dashes represent candidates, scaled by the normalised score. Green circle and dash represent the correct entry (when present), with the rank written adjacent.

The summary statistics for Categories 1 and 2 are presented in Table 1 and Table 2. Participants were allowed to enter updated submissions (resubmissions) following the competition deadline as Challenges 2, 4 and 6 suffered from a calibration issue that was only discovered after the submission deadline and Challenge 12 contained misleading noise peaks (for more details see [4]). These were not counted in declaring the winner (as the solutions had already been made public), but are included in the table below for completeness. The resubmitted statistics given in the tables include the original challenge results for challenges where no resubmissions were made. The original submissions for each participant are also displayed visually in Appendix B, Figures B1 to B6, including the rank of the correct compound, where applicable.

**Table 1:** Summary statistics for Category 1 by participant, before and after resubmission. Subm.: number of submissions; Cor(win): number of submissions with correct answer present and wins in brackets; *^r^* resubmitted results. Other abbreviations: see text. Wins given for original submissions only.

Participant	Subm.	Cor(win)	Avg. Rank	Avg. TC	Avg. BC	Avg. RRP	Avg. wRRP	Avg. *s̃*
Dunn *et al.*	11	9(9)	1.11	1.4	0.1	0.500	0.926	0.889
Shen *et al.*	14	8(3)	2.88	11.1	1.9	0.719	0.670	0.168
Dührkop *et al.*	14	8(5)	2.25	128.8	1.3	0.992	0.625	0.602
Dührkop *et al.* *^r^*	14	12	5.75	134.7	4.7	0.883	0.423	0.406
Neumann *et al.*	13	9(5)	4.33	639.1	3.3	0.991	0.565	0.561
Neumann *et al.* *^r^*	14	12	4.83	1915.0	3.8	0.997	0.508	0.434
Meringer *et al.*	14	11(9)	4.45	34.4	2.7	0.847	0.759	0.275
Meringer *et al.* *^r^*	14	14	3.29	36.6	1.7	0.941	0.885	0.226

**Table 2:** Summary statistics for Category 2 by participant, before and after resubmission. ^*r*^ resubmitted results. Abbreviations: see Table 1 and text.

Participant	Subm.	Cor(win)	Avg. Rank	Avg. TC	Avg. BC	Avg. RRP	Avg. wRRP	Avg. *s̃*
Dunn *et al.*	11	3(2)	5.7	4.7	3.3	0.556	0.606	0.4294
Ruttkies *et al.*	14	9(2)	401.0	1618.9	138.4	0.813	0.547	0.0041
Ruttkies *et al.* *^r^*	14	14	319.7	1226.3	188.1	0.838	0.616	0.0069
Shen *et al.*	14	4(2)	5.5	19.4	4.3	0.614	0.520	0.1226
Gerlich *et al.*	14	11(5)	237.5	1631.2	236.5	0.882	0.864	0.0020
Gerlich *et al.* *^r^*	14	14	305.4	2878.1	304.3	0.873	0.862	0.0010
Oberacher	5	3(3)	1.0	1.2	0.0	−	1.000	1.0000
Meringer *et al.*	6	5(2)	23.4	307.7	22.4	0.470	0.457	0.0887
Meringer *et al.* *^r^*	6	6	29.2	258.5	28.2	0.551	0.535	0.0741

Although the results from internal participants for Categories 1 and 2 are included in this paper to give a wider overview of the methods available, the internal participants were *not* considered in declaring the winner for the CASMI contest (and would not have won any category if they had been included).

### 3.3. External Participants

The following paragraphs summarise the external participants and their results, counted in declaring the winner of CASMI. The information about the submissions was taken largely from the abstracts the participants provided with their entries; for more details see the articles prepared by the participants as part of this special issue [25,26,27,28].

**W. Dunn *et al.*** [25] entered both Category 1 and Category 2, using Workflows 1 and 2 of PUTMEDID-LCMS [32]. The accurate mass and isotope abundance pattern were used to generate one or more molecular formulas for the challenges in Category 1. In Category 2, automatic and manual searching for candidate structures was performed using the Kyoto Encyclopedia of Genes and Genomes (KEGG) database [33] and ChemSpider [34], in that order, followed by *in silico* fragmentation with MetFrag [35] version 0.9 and manual assessment to remove entries considered biologically unreasonable.

Dunn *et al.* were clear winners of Category 1, supported by the summary statistics presented in Table 1. Eight answers were correct in first place, one in second place and only two submissions did not contain the correct answer (Challenge 2, which had 30 ppm error in the original data and Challenge 13, where the one formula submitted was unfortunately incorrect—the correct formula was absent in the reference file applied in Workflow 2). The only 3 challenges this team did not enter were Challenges 11, 12 and 16, which showed non-standard ionisation behaviour. Although their average *RRP* appears poor (0.5), their average *TC* is 1.4 and the *RRP* is only defined when *TC* > 2.

The team was unable to maintain their momentum in Category 2, where they submitted entries for the same 11 challenges. The correct answer was present in only three of the 11 entries, but here they were quite successful and won two of these challenges. Challenge 1 was correct and ranked first (equal with Oberacher and Gerlich *et al.*), while for Challenge 5 the correct answer was ranked 4*^th^*, higher than the other three entries (ranks 5, 275 and 386). The entry for Challenge 14 was ranked 12*^th^*, behind Gerlich *et al.* (rank 1) but in front of two others (ranks 22, 39). The correct answer was missing in the remaining eight submissions, but Challenges 3 (*TS* =0.86) and 10 (*TS* = 0.84) were very close (see Figure 2) and Challenge 4 was also quite close (*TS* = 0.74). As this team have mainly metabolomics experience, it is not surprising that they were more successful for the first six (metabolomics) challenges, rather than the environmental challenges.

**H. Shen *et al.*** [26] entered Categories 1 and 2 using FingerID [36] to predict the structural fingerprints of the challenge data, which were then used to search KEGG [33]. Mass spectra from MassBank [37] were used as training data.

This team submitted entries for all challenges in Category 1, with the correct solution ranked 1 for three challenges and ranks between 3 and 5 for another five challenges. The correct answer was missing for the remaining six challenges. This was the only team to get the correct answer for Challenge 2 using the original data, despite the 30 ppm error. Again, this team was more successful for the metabolite challenges rather than the environmental challenges.

Shen *et al.* won two challenges in Category 2, with the answer for Challenge 2 in 1*^st^* place and Challenge 6 in 11*^th^* place, higher than the only other participant with the correct entry present (Gerlich *et al.* with rank 25). The correct answer had rank 5 for Challenges 1 and 5, for the latter only just behind the winner’s rank of 4. The correct entry was missing for the remaining 10 challenges, but they got close in two cases—Challenge 4 (*TS* = 0.91) and 10 (*TS* = 0.84). Since this team based their searches on KEGG, it is not surprising that they missed the answers for many challenges, especially Challenges 10–17.

**K. Dührkop *et al.*** [27] entered Category 1 with a command line version of SIRIUS, combining isotope pattern and fragmentation tree scores [38,39]. The PubChem Molecular Formula search [40] was used to search for a (de)protonated form of the compound, for molecules with an intrinsic charge a protonated form was also added with a lower score.

SIRIUS performed excellently for Challenges 10–17 with expected ionisation behaviour, with the correct solution in first place for 5 of these challenges. The non-standard ionisation and in-source fragmentation of Challenges 11, 12 and 16 lead to an incorrect precursor assignment and the correct answer was missing in these cases. For Challenges 11 and 16 the correct formula was almost present, but with the incorrect number of hydrogen—at rank 1 and 5, respectively. The results for Challenges 1–6 (TOF data) were less successful, with ranks 3, 8 and 2 for Challenges 1, 3 and 5, respectively. As this team used a hard cut-off of 5 ppm (which was the error margin originally quoted on the web), they missed the correct answer for Challenges 2, 4 and 6 in their original submissions. Using the recalibrated data, they obtained rank = 2 for these three challenges. Because the mass accuracy decreased after recalibration for Challenge 5, the rank was worse with the recalibrated data (29, compared with 2 previously). With the removal of interfering peaks in Challenge 12, they achieved a resubmission rank of 18 for this challenge. Interestingly, although Dührkop *et al.* improved their number of correct answers with their resubmission, the results for Challenges 5 and 12 had a negative impact on the overall statistics.

**H. Oberacher** [28] entered Category 2 using automated searches of 4 spectral libraries. MassBank [37] was searched for all MS types, METLIN [41] for MS/MS spectra. The MS/MS and Identity searches in the NIST database [42] were also used, as well as the MSforID search in the ‘Wiley Registry of Tandem Mass Spectral Data MSforID’ [43,44]. The reference spectra in the different libraries were obtained on a variety of instruments and analytical settings and are thus not always directly comparable with the challenge data.

Although only 5 submissions were made, three of these were single suggestions that were correct and thus the winner in those challenges (Challenges 1, 13 and 15). The correct solution was missing in the other two submissions, but Challenge 10 was very close (*TS* = 0.84; positional isomers—see above) and although Challenge 14 was not too far off (carbazole instead of 1H–benz[g]indole; *i.e.*, a rearrangement of the aromatic rings), *TS* = 0.39 indicates only a poor similarity according to the fingerprint we used. Overall, the results of Oberacher show the power of spectral library searching very well, when the compound is present in the library—as well as showing the disadvantages when the correct compound is not in the library.

Oberacher was a deserved winner of Category 2, with clearly the best average rank, only one fewer submission containing the correct answer than the other two (external) participants in this category and many fewer “misses”.

In light of the descriptions above and the summary statistics in Table 1 and Table 2, we hope the readers and participants will agree with us that:

**... the winner of Category 1 is ... Dunn *et al.***

**... the winner of Category 2 is ... Herbert Oberacher.**

### 3.4. Internal Participants

Although the colleagues of the organisers of CASMI could not take part in the contest officially, we took the opportunity to evaluate our approaches on the challenges as well.

We did our best to approach the challenges objectively and did not optimise the parameters or scoring intentionally to improve the results. However, as our institutes are the sources of the challenges, our groups obviously have experience with these compounds and it was difficult to be completely objective. As for the external participants, the information is summarised below and in Table 1 and Table 2, while details are described in separate articles as part of this special issue [29,30].

**S. Neumann *et al.*** entered Category 1 using a small script to extract the isotope patterns from the MS peak lists for processing using Rdisop [7], based on the disop library [8]. No efforts were made to detect [M+H]^+^ or adducts as this script was only designed to test the evaluation scripts.

Originally, submissions were made for 13 of the 14 challenges, with 5 entries containing the correct solution ranked first, one in second place and three others with the correct solution ranked between 5 and 18. Five entries were missing the correct solution, while no submission was made for Challenge 16. The resubmitted entries were more successful, with six number one ranks and only two challenges missing the correct solution.

**M. Meringer and E. Schymanski** [30] entered all categories with different MOLGEN programs. MOLGEN–MS/MS [45] was used to enter Category 1, using a combined match value calculated from the MS isotope pattern match and MS/MS subformula assignment. MOLGEN 3.5 and 5.0 [46,47] were used for Category 2, with substructure information taken from fragmentation patterns and consensus scoring [48] combining *in silico* fragmentation results from MetFrag [35] with steric energy calculations from MOLGEN–QSPR [49]. MOLGEN–MS [50,51] was used for Categories 3 and 4, augmented with substructure information from the NIST database [42]. Category 4 was scored by combining MOLGEN-MS fragmentation, MOLGEN–QSPR steric energy and, where applicable, partitioning behaviour calculated using EPI Suite*^TM^* [52] in a consensus approach [48].

All challenges were entered for Category 1, with the correct solution ranked first for 6 challenges, three ranked 4*^th^*, two lower results (8, 23) and three missing the correct solution. As for the SIRIUS submissions, this was due to the incorrect 5 ppm error margin. Resubmissions for Challenges 1–6 resulted in a total of 9 number 1 ranks, 3 placed 4*^th^* still and two lower ranks (11, 14). In the end this resulted in more number 1 ranks than the CASMI winner, Dunn *et al.*, but a poorer average rank (3.29, compared with 1.11 for Dunn *et al.*).

Submissions were made for 6 of the 14 challenges in Category 2. The correct answer was present in all entries except one in the first round, with ranks between 3 and 63; two of these entries were also the best ranks for these challenges (Challenge 10, 11). These results are very good for structure generation approaches, but are “best case” results due to the prior experience of the participants with these and similar compounds. The correct answer was missing for Challenge 17 due to an incorrect substructure and was rectified after the submission deadline, where the correct answer was present at rank 58. Although these results are purely indicative and did not run in the competition, they do show that identification with structure generation is feasible with sufficient substructure information.

**C. Ruttkies *et al.*** [29] entered all challenges in Category 2 using MetFrag [35]. The peak lists were merged and converted into query files containing the exact mass of the precursor ions, which were deduced from the LC–HRMS/MS challenge data using simple heuristics. This caused incorrect candidate lists for Challenges 11 and 12, which were resubmitted later with the correct precursor information. Candidates were obtained from a local PubChem mirror (snapshot from September 2010), with an exact mass window of 5 ppm (except for the resubmission of Challenge 5, where 10 ppm was used), which meant that the correct candidate was missing from Challenges 2, 4 and 6 initially. For Challenges 10–17 the MetFrag score was used alone, while the Metabolite Likeness [53] was also included in the scores for Challenges 1–6. For the resubmission, an InChI Key filtering was employed to remove duplicate candidates and stereoisomers.

MetFrag had the highest average rank of all participants (320 following resubmissions—see Table 2), mainly due to the large candidate numbers that were not filtered manually. For the original submissions, MetFrag had the best ranked correct answer for two of the challenges (3 and 17) and was thus on par with three of the other participants who also had two “wins”. For Challenge 3, MetFrag was the only entry with the correct structure present (see Figure 1), while MetFrag outperformed MetFusion (see below) for Challenge 17 despite retrieving more candidates for this challenge. Following resubmissions, the correct solution was present in all MetFrag entries.

**M. Gerlich *et al.*** [29] entered Category 2 with MetFusion [14]. Submissions were made for all challenges. The peak lists were preprocessed as for MetFrag above, except that candidates were obtained from PubChem online with a default exact mass window of 10 ppm and an additional InChI Key filtering to remove duplicate candidates and stereoisomers. MassBank [37] was searched for ESI spectra: following resubmission also including APCI and APPI spectra. All calculations were performed using the command line version of MetFusion. As for MetFrag, the correct answer was missing for two challenges initially due to an incorrect precursor mass, while for Challenge 3 the correct solution was missing due to an incomplete PubChem query.

In the original submissions, MetFusion had the lowest rank for five challenges (1, 4 and 14–16); three of these with the correct structure also ranked 1*^st^*. Two of these (Challenges 1 and 15) were equal with Oberacher; Challenge 1 also with Dunn *et al*. After resubmission, three challenges had the correct answer ranked 1*^st^* (Challenges 1, 13 and 14). The average *RRP* of 0.873 (following resubmissions) is quite good and the highest of all participants, but the average rank of 305 means that many candidates still achieve better scores than the correct candidate in most cases. This is supported by the very low normalised score values. Altogether, MetFusion would theoretically have won seven challenges after resubmission. The details are given in [29].

## 4. Results by Challenge

In this section we present various statistics of CASMI 2012 by challenge, so readers can judge the various evaluation measures, the difficulty of the challenges and the success of the different strategies for themselves.

### 4.1. Statistics for Category 1

Summary statistics for Category 1 by challenge are shown in Table 3. This table clearly shows which challenges were relatively easy for the participants and which were more challenging. The results for Challenge 2 (with recalibrated data for most participants) are quite surprising: despite the highest mass, and thus, more possible candidate formulas within the error parameters given, the average rank of 1.3 is a fantastic result. Overall, following resubmission all submissions contained the correct answer for 7 challenges (1, 4–6, 10, 14–15), although only two of these had an average rank of 1.0 and could thus be termed “easy”. Surprisingly for the molecular formula calculation, the average *TC* is very large, ranging from 10.8 for Challenge 14 to 2931 for Challenge 2. These values are driven largely by the contributions of Neumann *et al.*, where no additional heuristics (such as nitrogen rule, double bond equivalents *etc.*) were used to filter the candidate formulae, and Dührkop *et al.*, to a lesser extent. This demonstrates that with so few participants, the averages are heavily weighted by individual contributions and we do not wish to over-interpret the results here.

**Table 3:** Summary statistics for Category 1 by challenge, using resubmitted entries where available. Chal. = Challenge Number; Subm. = Number of submissions; Correct = Number of submissions with correct answer present. Avg. = average. Other abbreviations: see text.

Chal.	Subm.	Correct	Avg. Rank	Avg. TC	Avg. BC	Avg. RRP	Avg. wRRP	Avg. *s̃*
1	5	5	7.4	906.0	6.4	0.761	0.388	0.221
2	5	4	1.3	2931.2	0.3	1.000	0.764	0.228
3	5	4	10.5	802.0	9.5	0.944	0.455	0.253
4	5	5	1.8	143.6	0.8	0.958	0.546	0.175
5	5	5	7.6	142.8	6.4	0.679	0.505	0.250
6	5	5	2.0	319.0	1.0	0.755	0.605	0.236
10	5	5	1.0	29.4	0.0	1.000	1.000	0.718
11	4	1	4.0	53.3	0.0	0.700	0.400	0.200
12	4	3	10.0	229.5	8.0	0.850	0.213	0.042
13	5	3	1.0	89.8	0.0	1.000	1.000	0.714
14	5	5	1.0	10.8	0.0	1.000	1.000	0.801
15	5	5	2.0	18.0	1.0	0.797	0.675	0.540
16	4	1	4.0	294.8	1.0	0.895	0.749	0.082
17	5	4	1.0	21.4	0.0	1.000	1.000	0.857

### 4.2. Statistics for Category 2

Summary statistics for Category 2 by challenge are shown in Table 4. The correct answer was present in all submissions for only two of the challenges, Challenge 1 (kanamycin A) and 5 (reticuline). Kanamycin A is well-represented in databases, including the relatively small KEGG Compound database, but the compound would be difficult for structure generation approaches due to the many possible substitution isomers on the three-ring system (11 substituents and 4 different groups). Reticuline is also present in KEGG and has quite a distinctive substructure that is also present in the spectrum (see [4], Figure A5) but the spectrum itself is very noisy and the distinctive peaks do not clearly dominate in intensity, such that it is very surprising that this challenge was so successful. Challenges 13–15, with clear and distinctive fragmentation patterns (see Figures A10–A12 in [4]) were quite successful, with the correct answer present in 4 of 6 submissions for all these challenges. No external participant found the correct solution for six challenges (3, 4, 11, 12, 16 and 17). Unsurprisingly Challenges 12 and 16, which caused difficulties in Category 1 already, had the fewest submissions, with only 3 submissions (including one external participant). Only four challenges had an average *RRP* > 0.9 (Challenges 2, 3, 12 and 17), while three of these four (*i.e*., not Challenge 12) also had *wRRP* > 0.9. Interestingly, these challenges had relatively poor normalized scores s̃; the challenges with the highest average *s̃* (Challenges 1, 2, 5, 13 and 15) were also those with the lowest average rank, which makes the normalised score an interesting metric to assess the scoring success.

**Table 4:** Summary statistics for Category 2 by challenge, using resubmitted entries where available. Abbreviations: see Table 3 and text.

Chal	Subm.	Correct	Avg. Rank	Avg. TC	Avg. BC	Avg. RRP	Avg. wRRP	Avg. *s̃*
1	5	5	2.4	646.2	1.4	0.732	0.818	0.4166
2	4	3	2.7	221.5	1.7	0.996	0.975	0.1012
3	4	2	11.5	1012.0	10.5	0.994	0.931	0.0033
4	4	2	264.0	1617.3	263.0	0.890	0.741	0.0006
5	4	4	668.3	1798.0	664.3	0.630	0.472	0.0842
6	4	3	105.7	2014.5	104.3	0.773	0.641	0.0190
10	6	3	434.7	287.2	427.3	0.391	0.190	0.0022
11	4	3	65.3	487.3	56.7	0.858	0.816	0.0458
12	3	2	86.0	1783.3	83.5	0.971	0.815	0.0045
13	6	4	2.5	736.5	0.8	0.777	0.865	0.3149
14	6	4	16.0	121.7	13.5	0.723	0.565	0.0227
15	6	4	50.3	877.2	48.5	0.700	0.712	0.2579
16	3	2	1649.5	2129.3	766.0	0.574	0.340	0.0001
17	5	3	53.7	727.4	52.7	0.960	0.911	0.0035

## 5. Discussion

The results for Category 1 show that molecular formula assignment is perhaps not as easy as often thought. Although all participants used more sophisticated methods than pure accurate mass assignment, only four of 14 challenges had an average rank of 1. Even the combination of isotope patterns and MS/MS information in the automatic approaches of Dührkop*et al.* and Meringer *et al.* was insufficient to define the correct candidate on the top place in many cases. Many of the challenges offered were quite large (5 > 350 Da) and did not have specific isotope information. The method of Dunn *et al.*, combining automated searches with expert knowledge, was unbeatable and shows that it is vital to incorporate additional information into the molecular formula selection already.

The results for Category 2, exemplified in the statistics in Table 4, confirm the general opinion about structure elucidation via MS. Identification efforts are generally very successful when the compound is present in a spectral library (especially if the spectrum is from a similar instrument), as proven by the success of Oberacher’s approach, but elucidation becomes more challenging as soon as no related compounds are present in a spectral library. Automated approaches such as MetFrag and MetFusion mean that users can retrieve many candidates from compound databases, but it is plain to see from the very large candidate numbers (average *TC* ≥ 1000 for both) that the scoring would have be phenomenally selective to have a chance; the average ranks of 305 (MetFusion) and 320 (MetFrag), both following resubmission, show that this is not yet the case. However, both achieved the most entries with the correct candidate present of all participants, even before resubmission. The contestants that used methods based on the smaller KEGG database, Dunn *et al.* and Shen *et al.*, appeared less successful as they missed the correct solution more often, but had much better ranks in the case where the correct molecule was in KEGG. Their average ranks of 5.5 (Shen *et al.*) and 5.7 (Dunn *et al.*) show that if sufficient information is available for their methods, they were much closer to the success of Oberacher than the compound database searching or structure generation approaches. Their results also show the advantage of using specialised databases in the right context. While structure generation can help in the case of very specific substructure information, this is not yet automated for MS/MS and novel approaches such as the maximum common substructure approach can have unexpected pitfalls for compounds with many possible substitution patterns. Thus, expert and sophisticated interpretation techniques are still essential for structure elucidation via MS/MS and fully-automated strategies have a long way to go before they can be applied routinely without extensive interpretation or post-processing of the results.

## 6. Conclusions and Perspectives

This was the first CASMI contest; it was a pleasure and a privilege to organise it and receive submissions from such high quality research groups. Despite what appears to be low participant numbers, it spurred a lot of interest and discussions, including those with colleagues who were unable to find the time to participate. The results from the participants show that the current state-of-the-art in identification requires an automated approach combined with expert knowledge for the molecular formula (*i.e*., isotope patterns and MS/MS fragmentation are insufficient), while database searching is unbeatable for structure identification *where the structure is present in a spectral library*. Automated methods alone are still unlikely to rank the correct structure among the top candidates without significant input of expert knowledge. However, such automated approaches are required for higher throughput routine annotation of MS data in biological applications, non-targeted screening in the environmental sciences and other fields.

The next CASMI will be coordinated in 2013/14 by T. Nishioka and a team of Japanese mass spectrometrists. We are willing to support them with suggestions based on our experience. The evaluation measures presented here provided a good overview of the strengths of individual approaches, while using the absolute rank to declare the winner proved to be both simple and most realistic for real identification challenges. The introduction of a new contest category could be considered, where a list of candidates is provided along with spectral data for the participants to rank using their methods. This may improve the comparability between the different approaches.

The organising team of CASMI 2012 look forward to participating in the next CASMI and hope to see many more participants to support this initiative of providing open data to allow the evaluation of independent methods on consistent data.

## Appendix

### A. Summary of CASMI 2012 Challenges

**Table A1:** LC Challenges for CASMI 2012.

Challenge	Trivial Name	Formula
1	Kanamycin A	C_18_H_36_N_4_O_11_
2	1,2-Bis-O-sinapoyl-beta-d-glucoside	C_28_H_32_O_14_
3	Glucolesquerellin	C_14_H_27_NO_9_S_3_
4	Escholtzine	C_19_H_17_NO_4_
5	Reticuline	C_19_H_23_NO_4_
6	Rheadine	C_21_H_21_NO_6_
10	1-Aminoanthraquinone	C_14_H_9_NO_2_
11	1-Pyrenemethanol	C_17_H_12_O
12	alpha-(o-Nitro-p-tolylazo)acetoacetanilide	C_17_H_16_N_4_O_4_
13	Benzyldiphenylphosphine oxide	C_19_H_17_OP
14	1H-Benz[g]indole	C_12_H_9_N
15	1-Isopropyl-5-methyl-1H-indole-2,3-dione	C_12_H_13_NO_2_
16	1-[(4-Methoxyphenyl)amino]-1-oxo-2-propanyl-6-oxo-1-propyl-1,6-dihydro-3-pyridazinecarboxylate	C_18_H_21_N_3_O_5_
17	Nitrin	C_13_H_13_N_3_

The challenges for CASMI 2012 Categories 1 and 2 (LC–HRMS/MS) are shown in Table A1. A table with structures as well as PubChem and ChemSpider identifiers is available in [4] and on the CASMI website [3], along with a CSV file for download.

### B. Participant Submissions by Score

In Figures B1 to B6 we present plots of the *original* participant submissions for Category 2 with the grey dash representing each candidate scaled by the normalised score. The correct answer (where present) is circled and marked with a dark green dash; the rank of the correct answer is written next to this dash. These figures are a useful visualisation of the results.
